# Growth mixture modelling of HIV-exposed uninfected children under five in sub-Saharan Africa

**DOI:** 10.21203/rs.3.rs-10868788/v1

**Published:** 2026-10-22

**Authors:** Mthobisi Mxolisi Zondi, Henry G Mwambi, Sileshi Fanta Melesse, Nonhlanhla Yende-Zuma

## Abstract

Antiretroviral therapy scale-up has reduced vertical HIV transmission, expanding the population of HIV-exposed uninfected (HEU) children in sub-Saharan Africa. However, heterogeneity in their growth patterns and the influence of country context and maternal factors remain poorly understood. Longitudinal height-for-age (HAZ) and weight-for-age (WAZ) z-scores from HEU children (birth-60 months) in the multi-country PROMOTE cohort (Malawi, South Africa, Uganda, Zimbabwe) were derived. Growth mixture modelling with fractional polynomials was implemented using the Bolck-Croon-Hagenaars (BCH) method, incorporating country as a predictor, and maternal and household factors were examined in weighted multinomial models. Three distinct growth trajectories were identified for HAZ: severe stunting, 43.9%; mild-to-moderate, 39.9%; and adequate, 16.2%; and for WAZ: severe underweight, 23.6%; stable mild, 68.2%; and recovery, 8.1%. Moderate entropy (HAZ = 0.555; WAZ = 0.538) supported BCH weighting. Compared with Malawian children, South African children had significantly lower odds of severe underweight (aOR 0.57, 95% CI 0.37–0.88) and higher odds of being in the WAZ recovery trajectory (aOR 4.96, 95% CI 1.89–13.01). In multivariable analyses, private access to a flush toilet, maternal formal employment, and household electricity were associated with more favourable growth trajectories. Compared with PI-based regimens closer to delivery, EFV/NVP-based (aOR 0.44, 95% CI 0.27–0.73) and DTG-based (aOR 0.52, 95% CI 0.33–0.84) were associated with lower odds of mild-to-moderate stunting relative to adequate growth. Among HEU children, growth trajectories were more consistently associated with household infrastructure and country context than with maternal HIV disease severity. However, ART regimen type showed notable associations with growth trajectory membership, though these findings require confirmation in future research. These findings support the integration of social and structural interventions alongside ART programmes.

